# Supplementary material for: Implementing inclusive online psychological support after stroke: professional stakeholder views from a qualitative study
Source: BMC Health Serv Res. 2026 Jun 6;26:1111. doi: 10.1186/s12913-026-14816-4 (PMC13471390; doi:10.1186/s12913-026-14816-4)
Supplement: Supplementary file 2 [file 12913_2026_14816_MOESM2_ESM.pdf]

Appendix B. Interview Schedule – mapped to CFIR and NPT Domains

Questions were informed by both the Consolidated Framework for Implementation Research (CFIR) (Nevedal et al, 2021) and Normalization Process Theory (NPT) (May, 2009; Murray, 2010).

The table below summarises the questions (and probes) for each participant type and how they map onto CFIR / NPT domains:

- Group 1: Commissioning managers
- Group 2: Service managers
- Group 3: Clinicians

		**Asked of Group**				
**Question**	**Probes**	**1**	**2**	**3**	**CFIR Domain(s)**	**NPT Construct(s)**
WAterS-2 is about offering inclusive, accessible support to help people psychologically adjust to stroke and *prevent* a mental health crisis. How does this align with your commissioning goals?	Could you tell me about any services that are commissioned across geographical boundaries?<br><br>If not a good fit, explore alternative services that may be better suited?	✓	✓		• Intervention Characteristics<br>• Outer **and** Inner Setting;<br>• Characteristics of Individuals	• Coherence<br>• Collective Action
What factors influence the adoption of new services in your area?	What evidence or guidelines do you look for to commission / adopt services or interventions, such as WAterS-2?<br><br>How do influential voices and leadership within your organisation influence the adoption of new interventions?<br><br>Resource / cost considerations and how they are calculated?	✓	✓		• Process<br>• Outer **and** Inner Setting<br>• Intervention characteristics	• Collective Action<br>• Coherence

Question	Probes	1	2	3	CFIR Domain(s)	NPT Construct(s)
Can you tell me about any instances when you have commissioned/ provided interventions targeted at minoritized ethnic groups?	Do you have any thoughts on how interventions are made suitable for these groups and/or any suggestions for us when seeking expertise in including minoritized ethnic communities locally?<br><br>Are there existing initiatives focused on equity and inclusion that could apply to WAterS-2?	✓	✓		• Process<br>• Outer **and** Inner Setting	• Collective Action<br>• Cognitive Participation
Tell me about any strategies you already use to support minoritized ethnic communities in accessing and using post-stroke services?	Do you have any thoughts on how interventions are made suitable for these groups and/or any suggestions for us when seeking expertise in including minoritized ethnic communities locally?<br><br>Are there existing initiatives focused on equity and inclusion that could apply to WAterS-2?		✓	✓	• Outer setting<br>• Process<br>• Characteristics of Individuals	• Cognitive Participation<br>• Collective Action
What about strategies for supporting people with aphasia to access?	(same prompts)		✓	✓	• Process<br>• Characteristics of Individuals	• Cognitive Participation<br>• Collective Action
If you implemented WAterS, what information would you want to collect to evaluate its success?	Any key performance indicators we should be aware of?<br><br>What would 'success' look like, in your opinion?	✓	✓		• Intervention characteristics<br>• Process	• Reflexive Monitoring

Question	Probes	1	2	3	• CFIR Domain(s)	• NPT Construct(s)
Considering the information received prior to the interview, which frontline staff might be best positioned to deliver WAterS-2?	Do they know about Acceptance and Commitment Therapy?<br><br>How is staff time currently allocated? How do you release and compensate staff for training and intervention delivery?<br><br>What might motivate staff to be part of WAterS-2?		✓	✓	• Inner Setting	• Collective Action
If you implemented WAterS-2, how would you identify suitable stroke survivors to receive?	Are there differences for reaching people from different demographics (e.g., minoritized ethnic communities / people with aphasia)?		✓	✓	• Process	• Cognitive Participation<br>• Collective Action
What experience does your organisation have with videoconferencing tools like Zoom or Teams for staff training and service provision?	How do you find using remote technologies?		✓	✓	• Inner setting	• Collective Action
Overall, what do you think are the main challenges to delivering WAterS-2?	How might these barriers be mitigated?	✓	✓	✓	*Answer dependent? Could be all*	*Answer dependent? Could be all*
Overall, what do you think could be the biggest enablers for delivering WAterS-2 in your service?		✓	✓	✓	*Answer dependent? Could be all*	*Answer dependent? Could be all*

Question	Probes	1	2	3	• CFIR Domain(s)	• NPT Construct(s)
WAterS-2 is informed by Acceptance and Commitment Therapy. Do you or your colleagues have much experience with this therapy?	What further information would you want to receive to help make an informed decision about delivering WAterS-2?<br><br>What further training or support might you want to deliver WAterS-2?			✓	• Characteristics of Individuals	• Collective Action
Is there anything else you'd like to say about implementing WAterS-2?		✓	✓	✓	n/a	n/a

References

- May C and Finch T. Implementing, Embedding, and Integrating Practices: An Outline of Normalization Process Theory. Sociology. 2009; 43: 535-54.
- Murray et al. Normalisation process theory: a framework for developing, evaluating and implementing complex interventions. BMC Medicine. 2010; 8 (63). https://doi.org/10.1186/1741-7015-8-63
- Nevedal, A.L.,et al. Rapid versus traditional qualitative analysis using the Consolidated Framework for Implementation Research (CFIR). Implementation Sci 16,67(2021). https://doi.org/10.1186/s13012-021-01111-5
